# Isolation of Swine Bone Marrow Lin-/CD45-/CD133 + Cells and Cardio-protective Effects of its Exosomes

**DOI:** 10.1007/s12015-022-10432-x

**Published:** 2022-08-04

**Authors:** Hongxiao Li, Jianjun Gu, Xiaolin Sun, Qisheng Zuo, Bichun Li, Xiang Gu

**Affiliations:** 1 Medical College of Yangzhou University, Yangzhou, 225001 Jiangsu China; 2 Department of Cardiology, Northern Jiangsu People’s Hospital, Yangzhou, 225001 Jiangsu China; 3 College of Animal Science and Technology, Yangzhou University, Yangzhou, 225001 Jiangsu China

**Keywords:** CD133 + /Lin-/CD45- cells, Swine bone marrow, Isolation, Exosomes, Cardiac fibrosis

## Abstract

**Background:**

The identification in murine bone marrow (BM) of CD133 + /Lin-/CD45- cells, possessing several features of pluripotent stem cells, encouraged us to investigate if similar population of cells could be also isolated from the swine BM. Heart failure is the terminal stage of many cardiovascular diseases, and its key pathological basis is cardiac fibrosis (CF). Research showed that stem cell derived exosomes may play a critical role in cardiac fibrosis. The effect of exosomes (Exos) on CF has remained unclear.

**Objective:**

To establish an isolation and amplification method of CD133 + /Lin-/CD45- cells from newbron swine BM in vitro, explore an highly efficient method to enrich swine bone marrow derived CD133 + /Lin-/CD45- cells and probe into their biological characteristics further. Furher more, to extract exosomes from it and explore its effect on CF.

**Methods:**

The mononuclear cells isolated from swine bone marrow by red blood cell (RBC) lysing buffer were coated by adding FcR blocking solution and coupled with CD133 antibody immunomagnetic beads, obtaining CD133 + cell group via Magnetic Activated Cell Sorting (MACS). In steps, the CD133 + /Lin-/CD45- cells were collected by fluorescence-activated cell sorting (FACS) labeled with CD133, Lin and CD45 antibodies, which were cultured and amplified in vitro. The biological features of CD133 + /Lin-/CD45- cells were studied in different aspects, including morphological trait observed with inverted microscope, ultrastructural characteristics observed under transmission electron microscope, expression of pluripotent markersidentified by immunofluorescent staining and Alkaline phosphatase staining.

The Exos were extracted using a sequential centrifugation approach and its effects on CF were analyzed in Angiotensin II (Ang-II) induced-cardiac fibrosis in vivo. Rats in each group were treated for 4 weeks, and 2D echocardiography was adopted to evaluate the heart function. The degree of cardiac fibrosis was assessed by Hematoxylin–Eosin (HE) and Masson's trichrome staining.

**Results:**

The CD133 + /Lin-/CD45- cells accounted for about 0.2%-0.5% of the total mononuclear cells isolated from swine bone marrow. The combination of MACS and FACS to extract CD133 + /Lin-/CD45- cells could improved efficiency and reduced cell apoptosis. The CD133 + /Lin-/CD45- cells featured typical traits of pluripotent stem cells, the nucleus is large, mainly composed of euchromatin, with less cytoplasm and larger nucleoplasmic ratio, which expressed pluripotent markers (SSEA-1, Oct-4, Nanog and Sox-2) and alkaline phosphatase staining was positive.Animal experiment indicated that the cardiac injury related indexes (BNP、cTnI、CK-MB and TNF-α), the expression of key gene Smad3 and the degree of cardiac fibrosis in Exo treatment group were significantly reduced compared with the control group. 4 weeks after the treatment, cardiac ejection fraction (EF) value in the model group showed a remarkable decrease, indicating the induction of HF model. While Exo elevated the EF values, demonstrating cardio-protective effects.

**Conclusion:**

The CD133 + /Lin-/CD45- cells derived from swine bone marrow were successfully isolated and amplified, laying a good foundation for further research on this promising therapeutic cell. The Exos may be a promising potential treatment strategy for CF.

**Graphical Abstract:**

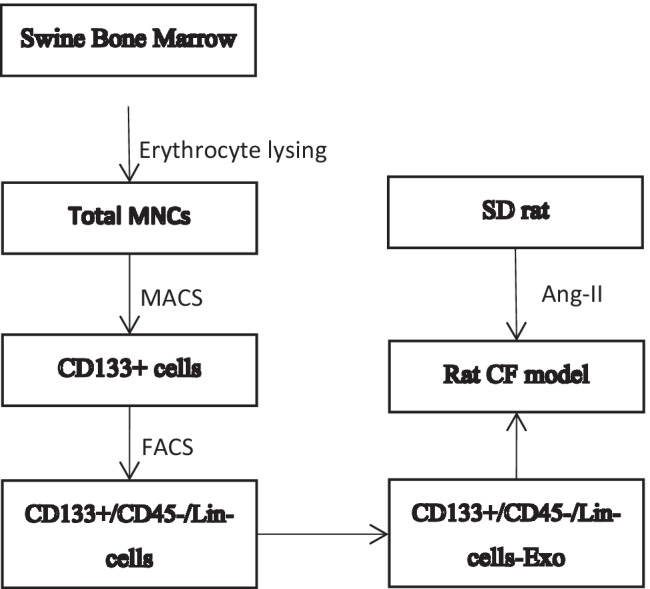

## Introduction

Heart failure is the terminal stage of various heart diseases. As its morbidity, mortality and re-hospitalization rate continues increasing, it’s called the "last battlefield of heart disease". According to the survey, the number of heart failure patients worldwide in 2017 exceeded 60 million [1].

The key pathological basis of heart failure is cardiac fibrosis (CF), in which pathological activation of cardiac fibroblasts and abnormal deposition of extracellular matrix lead to delayed conduction velocity of cardiac tissue, ultimately leading to reduced cardiac compliance and cardiac function [2]. At the present stage, the main treatments for heart failure include medication, cardiovascular implantable devices and heart transplantation. As medication and devices cannot effectively reverse CF, and heart transplantation is limited by the specificity of heart donor, the existing treatments do not achieve the expected efficacy [3]. Therefore, exploring new methods to reverse CF, improve cardiac remodeling and develop more effective treatments, is the most urgent task in the field of heart failure research at present.

One of the major interests about stem cells is their potential therapeutic applications [4]. Although there are still considerable dispute about stem cells therapy, the positive results obtained in the repair of damaged myocardium indicated it had been a promising candiadate [5]. In fact, diverse stem cells have been identified and applied to the regeneration medicine, including bone marrow-derived mononuclear cell (BM-MNCs) [6] and umbilical cord blood-derived stem cells (UCB-SCs) [7]. At the current state of stem cell clinical application, there is no convincing data showing the superiority of any tissue committed monopotent stem cells (TCSCs), so heterogeneous population of BM-MNCs is most commonly used [8]. Studies have shown pluripotent stem cells (PSCs) are precursors of TCSCs during organ/tissue rejuvenation and a source of these cells in emergency situations when organs are damaged (e.g., myocardial infarction or stroke) [9]. The application of PSCs has shown very encouraging results, including iPSCs and epiblast-like stem cells (ESCs) isolated from UCB.

Both pluripotent VSELs and iPSCs were reported in 2006. Though 2012 Nobel Prize was awarded to iPSCs technology, even today the authenticity of VSELs existence is still not extensively accepted by academic community, mainly due to the difficult isolation of VSELs [10, 11]. So far, most of these original studies are from Ratajczak’s group [12]. The independent validation of VSELs remains further investigation. The primary goal of our study was to establish the method of separation and culture of the BM CD133 + /Lin-/CD45- cells in vitro. Meanwhile we aimed to explore its optimized sorting process and observe the morphological structure and biological characteristics of swine VSELs from BM in vitro. We improved the protocol of isolating CD133 + /Lin-/CD45- cells based on Dr. Ratajczak's method [11] and other researcher’s failures he put forward [13]. We successfully isolated CD133 + /Lin-/CD45- cells from swine BM for the first time, laying a good foundation for further research on this promising therapeutic cell.

Previous studies have shown that stem cell transplantation has a certain therapeutic effect on CF [14], but MSCs after transplantation have deficiencies such as low survival rate and low homing size, and their safety is still unclear. Potential immune response and tumorogen risk limit the direct application of MSCs [15]. Several studies have shown that stem cells can promote the repair of damaged myocardial tissue by secreting exosomes (Exo) and cytokines [16]. Exo, as a carrier of nucleic acids, proteins and other small molecules, can participate in the regulation of cell proliferation, differentiation, apoptosis and other biological processes through molecular dialogue between cells. Moreover, compared with stem cells, Exo has better biocompatibility and lower immunogenicity, which can effectively avoid many defects in the clinical application of stem cells [17]. Previous studies have shown that Exo secreted by stem cells can inhibit CF and improve cardiac function [18, 19]. Shao et al. even found that Exo has a better effect on improving cardiac remodeling than stem cells [20]. Some scholars have proposed that Exo secreted by stem cells may be the main mechanism for the benefit of stem cell therapy [21, 22]. Then can Exo be used to replace stem cells in the treatment of CF? What are the specific components in Exo that are really involved in regulating CF repair? These issues need to be further explored and clarified.

In addition, when the stem cell is stimulated by the environment, the Exo contents (miRNA, lncRNA, protein, etc.) secreted by it will change due to chromatin remodeling, thus affecting its biological effects [23, 24]. Therefore, Exo can be used as a carrier for drug delivery system. Studies have confirmed that atorvastatin pretreated rat MSC source Exo can inhibit endothelial cell apoptosis, promote angiogenesis and reduce CF by delivering LncRNA H19 [25]. Therefore, drug intervention can change the content composition of stem cell Exo and enhance the therapeutic activity of Exo, and the development of alternative therapies combined with drugs and stem cell Exo has broad prospects for the clinical treatment of cardiovascular diseases.

## Materials and Methods

### Animals

Newborn swine (< 3d), coming from Yangzhou University Animal Center (Yangzhou, China), were kept under the conditions of temperature 21 ± 2℃. All swine were handled under sterile conditions and were maintained in germ-free isolators located in the Central Laboratory Animal Facilities of Yangzhou University, China. All animal procedures used were performed in accordance with the Guiding Principles for Care and Use of Experimental Animals[26]. The animal experiments were approved by institute stem cells and Animal Care Committees of Yangzhou University.

### Isolation of Bone Marrow Mononuclear Cells by Erythrocyte Lysis

The tibiae and femurs were removed from these newborn swine after being killed by intravenous injection of euthanasia drugs. The BM was flushed out from these bones. After cleaning to remove muscle and connective tissue, the remaining bone tissue specimens were washed twice with Ca^2+^ and Mg^2+^-free Dulbecco's Phosphate-Buffered Saline (PBS, Nacalai Tesque, Kyoto, Japan). Bone marrow mononuclear cells (BM-MNCs) were obtained following lysis of red blood cells with 1 × BD Pharm Lyse Buffer ( (BD Pharmingen, San Jose, CA, USA) for 10 min.

### Enrich CD 133 + Cells from BM-MNCs by MACS Sorting

After isolations, BM-MNCs were resuspended in FC receptor blocking (BD Biosciences) and incubated on ice for 30 min. Cells were co-stained for 30 min on ice in the dark with Anti-CD133 Magnetic Microbeads (Miltenyi Biotec). Wash cells with PBS and centrifuge at 400 × g for 10 min. Aspirate supernatant and resuspended in PB buffer. Prebalance the LS column (Miltenyi Biotec) with 3 ml PBS, apply cell suspension onto the column that has been fixed in the magnetic Separator (Miltenyi Biotec). Wash column with 10 ml PBS. Remove column from separator and flush out the magnetically labeled cells into 15 ml collection tube with 5 ml PBS. Centrifuge the magnetically labeled cells at 400 × g for 10 min. Subsequently, the pellet containing CD 133 + cells was resuspended in appropriate FACS buffer for further sorting.

### Flow Cytometric Analysis (FCM) and Fluorescence-Activated Cell Sorting (FACS)

The FCM analysis and FACS sorting were performed using a BD FACSCanto II flow cytometer (BD Biosciences) and a BD FACSAria cell sorter (BD Biosciences). Prepurified fractions of cells that were obtained after MACS in RPMI 1640 medium supplemented with 2% FBS with antibodies against CD133, CD45, and Lin markers. All samples were washed by adding 3 ml of RPMI 1640 medium with 2% FBS and centrifuging 10 min at 500 × g, 4 ℃. Then cells were resuspended for sorting in RPMI 1640 medium with 2% FBS and transferred to new 5 ml round-bottom tubes passing through a 40-μm strainer/mesh filter to remove cell clumps. 4 ml culture medium were added into tube and keep on ice until analysis and sorting.

Set the FSC and SSC parameters in logarithmic or linear scale and the threshold on the FSC parameter. Run the mixture of predefined-sized microspheres (size calibration beads with standard diameters of 1, 2, 4, 6, 10, and 15 μm) and adjust the threshold for the machine to include all objects as small as 2-μm. Set up the minimal threshold to be able to see 2-μm beads. Set the gate, which will include all objects in the 2- to 10-μm size range on the dot-plot that shows objects according to their FSC and SSC parameters. Run the samples with stained BM-MNCs and adjust the mononuclear cell gate to include agranular objects of 2- to 10-μm in size.

Perform compensation calculations and prepare the logical gating strategy resulting in identification and isolation of CD133^+^/Lin^−^/CD45^−^ VSELs.

### Culture and Amplification of CD133 + /Lin-/CD45- Cells

The division and amplification of CD133 + /Lin-/CD45- cells were slow in the co-cultures with 10% FBS and RPMI 1640, maintaining the growth state of single cells. In order to promote the division and proliferation of CD133 + /Lin-/CD45- cells, porcine myoblastocytes were cultured with mitomycin C solution to produce feeding layers. Then the isolated CD133 + /Lin-/CD45- cells were cultured in culture plates with feeding layers of myoblastocytes, together with 10% FBS and RPMI 1640. During CD133 + /Lin-/CD45- cells expansion and proliferation, culture medium was replaced every three days and cells were passaged after they reached 70% confluency.

### Transmission Electron Microscopy Analysis

For transmission electron microscopy (TEM), the CD133 + /Lin-/CD45- cells were fixed in 3% glutaraldehyde in 0.1 M cacodylate buffer pH 7.4 for 10 h at 4℃, post-fixed in osmium tetride and dehydrated. Fixed cells were subsequently embedded in LX112 and sectioned at 800A˚, stained with uranyl acetate and lead citrate and viewed on a Philips CM10 electron microscope operating at 60 kV.

### Immunofluorescence Staining and Alkaline Phosphatase Staining

The CD133 + /Lin-/CD45- cells in healthy growth conditions were plated in 24-well plates with climbing slides. Adherent growth was detected after 24 h by immunofluorescence staining. The CD133 + /Lin-/CD45- cells were fixed with 4% paraformaldehyde at 4℃ for 30 min. Fixed cells were permeabilized with 0.1% Triton X-100 (Solarbio) and blocked with blocking solution (2% FBS/0.1% Tween 20 (Solarbio)). Fixed cells were incubated overnight at 4℃ with primary antibodies (1:200 dilution) against swine Nanog, Oct-4, and Sox2 (Proteintech, Chicago, IL, USA). Cells stained with diluent only served as the negative control. After overnight incubation, cells were washed with PBS three times for 5 min each, incubated with CY3-conjugated secondary antibody (1:500; Beyotime, Shanghai, China) for 1 h at room temperature, and washed five times with PBS. Nuclei were stained with 4’,6-diamidino-2-phenylindole (Solarbio). Slides were mounted and examined using a fluorescent microscope (TE2000 Nikon, Tokyo, Japan).

ALP activity is performed using ALP assay kit (Pars Azmoon, Iran) according to the manufacturer's protocol. VSELs cells were fixed in a DMEM/F12 medium for culture, rinsed with PBS three times. The ALP activity is assessed by measuring Alkaline phosphatase stain and its conversion. Once the working solution was discarded, the cells were washed with PBS again. Images were captured using a light microscope at a magnification of × 4. ALP activity assay was performed as previously reported [27].

### RNA Extraction and RT-PCR Analysis

RNA from cells was isolated using the SV Total RNA Isolation System (Promega, Germany), according to manufacturer's instructions. cDNA was synthesized from 1 μg total RNA using the QuantiTect Reverse Transcription Kit from Qiagen (Hilden, Germany). qRT-PCR was carried out using the QuantiFast Sybr Green PCR Kit (Qiagen, Germany) with 20 ng cDNA as template. Normal myoblast were used as control group. cDNA was amplified by PCR using a Qiagen PCR kit according to the manufacturer’s instructions. Nanog, Oct-4, Sox2, CD133, and CD45 expression was detected by fluorescent qPCR. The 20μL PCR amplification reaction included 2μL cDNA, 10μL SYBR Taq, 0.8μL forward primer, 0.8μL reverse primer, 0.4 μL RoxII, and 6μL double-distilled water. Each experimental condition was repeated in triplicate. Relative mRNA quantities were determined using the 2^−ΔΔCT^ method.

### Treatment of CD133 + /Lin-/CD45- Cells with 5-Aza

After overnight incubation, the medium was aspirated and cells were washed with PBS three times for 3–5 min. New culture medium with 10 μM 5-Aza (Sigma-Aldrich Co., St. Louis, MO, USA) was added and subsequently replaced every three days. After 5-AzaC treatment, cell morphology was observed daily using a phase contrast microscope (DMILPH1 Leica).

### Isolation and Identification of Exos

Exosomes were prepared using sequential centrifugation according to methods described previously. The exosomes were isolated from the supernatants of CD133 + /Lin-/CD45- cells using ExoQuick-TC Kit (System Biosciences, USA) in accordance with manufacturer’s instructions. The CD133 + /Lin-/CD45- cells were cultured in a conditioned medium containing 10% exosome-free fetal bovine serum (FBS) for 48 h for the preparation of exosomes isolation, and then the supernatants of CD133 + /Lin-/CD45- cells were collected by initial centrifugation at 3,000 g for 15 min to pellet and remove the cell debris, then followed by sequential centrifugations at 13,000 g for 30 min, followed by centrifugation at 100,000 g for 60 min (Himac CS150GXII, HITACHI, Japan). Then the exosome-enriched fraction was resuspended in phosphate-buffered saline (PBS). To identify the Exos, exosomes resuspended in 0.1 mL of PBS was used for a transmission electron microscope and particle size analysis, and exosomes resuspended in 0.1 mL RIPA buffer for protein quantification and Western blotting. 0.5 × 10^9^ exosomes were administered to cultured cells in vitro and 5 × 10^9^/40μL to experiment rats in vivo to evaluate the function of Exos. Besides, the morphologies of exosomes were observed with a transmission electron microscope (Hitachi H-7100 microscope; HITACHI, Japan).

### Cardiac Fibrosis Model Establishment and Exosomes Treatment

Healthy adult SD rats (weight 200-300 g) obtained from the experimental animal center of Yangzhou University (Yangzhou, China) were maintained in a constant temperature and humidity environment under specific pathogen-free conditions, with 12-h light/dark cycles and free access to food and water. Cardiac fibrosis model rats were established by subcutaneously injected with Angiotensin II (Ang-II, 1.44 μg/g/d) per day for 2 weeks. Meanwhile, the same volume of diluted acetic acid was given to the control group. Rats in treatment group were injected with 100 μg (100μL) Exos via pericardium puncture. Meanwhile, the same volume of PBS was given to rats in the control group. The exosomes or PBS injection was performed after the last Ang-II injection, and the injection lasts for 7 days. The tissue of the heart was harvested 14 days after the exosomes or PBS treatment. All rats were deeply anesthetized with sodium urethane intraperitoneal injection and perfused transcardially with PBS containing heparin. Then, 4% of paraformaldehyde was used to fix the brain tissues at 4℃, pH 7.4. Hearts were removed from the body and postfixed in 4% paraformaldehyde for 24 h. The extracted heart tissue was dehydrated in a gradient sucrose solution (10%, 20%, and 30%).

### Hematoxylin–Eosin (H&E) and Masson Staining

The left ventricles of the rat hearts were fixed in 4% paraformaldehyde and embedded in paraffin for H&E and Masson staining. The hearts were fixed for 24 h in 4% paraformaldehyde, then the tissue blocks of heart were dehydrated, embedded in paraffin. Finally, the left ventricles of hearts were cut into 4-μm-thick slices for staining. The prepared slices of heart were heated overnight at 37℃, then dewaxed, and stained with H&E and Masson trichrome according to the standard procedures.

### ELISA Test of Cardiac Injury and Western Blot Analysis of Fibrosis Proteins

The levels of myocardial injury indicators BNP, CK-MB, cTnI and TNF-α were detected by ELISA in venous blood samples during the model manufacturing stage and before and after Exo treatment. The expression level of Samd3 in the left ventricle of rats 14 days after treatment with exosomes or PBS was quantified by Western blotting. β-actin was used as an internal control to demonstrate equal protein loading. All samples were performed in duplicate. The software of Image Lab 3.0 system (Bio-Rad, USA) was used for quantification the densities of bands. Densitometry analysis was carried out using ImageJ.

### Echocardiographic Assessment of Left Ventricular Function

Echocardiography was conducted on the 28th day after operation using Vevo2100 ultrasound (VisualSonics, Canada) and corresponding probe (MS-250) with the center frequency of 21 MHz. The left ventricle's two-dimensional M-mode images of the left parasternal short axis sections were obtained. On these section images, three cardiac cycles on every measure point were recorded. Left ventricular end-systolic diameter (LVEDs), left ventricular end-diastolic diameter (LVEDd) and EF were measured and collected.

### Statistical Analysis

Data obtained were presented as means ± standard error. Statistical significance was determined using the SPSS 23 statistical program. Variance tests were performed on the data, and GraphPad Prism 7.0 software was used for graphing. The criteria for significance were defined as ∗ *p* < 0.05, ∗  ∗ *p* < 0.01, and ∗  ∗  ∗ *p* < 0.001.

## Results

### Isolation and Purification of CD133 + /Lin-/CD45- Cells

**Fig. 1:**
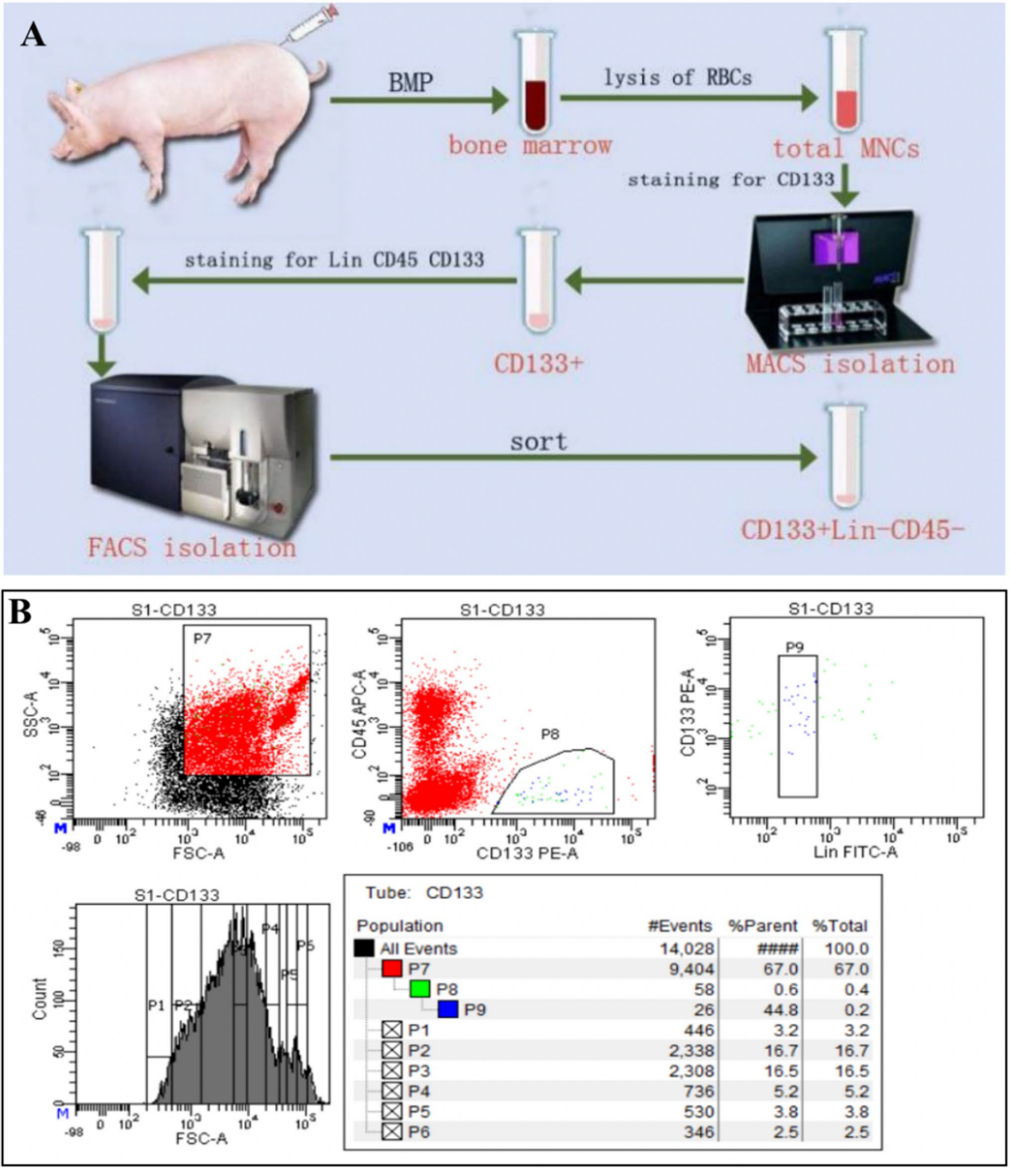
The isolation and purification process of swine BM CD133 + /Lin-/CD45- cells. **A**. The tibiae and femurs were removed from the newborn swines and BM was flushed out from the bones. The mononuclear cells isolated from swine BM by red blood cell lysing buffer were coated by adding FcR blocking solution and coupled with CD133 antibody immunomagnetic beads, obtaining CD133^+^ cell group via MACS. In steps, the CD133^+^/Lin^−^/CD45^−^ VSELs were collected by FACS labeled with CD133, Lin and CD45 antibodies, which were cultured and amplified in vitro. **B**. Gating strategy for identification of CD133^+^/Lin^−^/CD45^−^ populations by classical FACS. Total nucleated cells (TNCs) derived from BM were stained for CD45, Lin, CD133 and further analyzed by LSR II (Becton Dickinson). BM cells are visualized on dot-plot showing FSC versus SSC signals, which are related to the size and granularity/complexity of the cell, respectively. Total 1 × 10^4^ of analyzed TNCs is displayed in this dot-plot to visualize the population distribution. Objects from region P7 (CD133 +) were further analyzed for CD45 markers expression and only CD45^−^ events were included into region P8. Lower, right dot-plot cells stained for Lin and analyzed for Lin expression. CD45 − /CD133 + /Lin − (region P9) were further “back-gated”on FSC versus SSC dot-plot to visualize cell size distribution. Percentages show content of each subpopulation among TNCs in representative sample

By employing erythrocyte lysis, MACS and FACS successively, We could accurately separate CD133 + /Lin-/CD45- cells without contamination of hemopoietic stem cell (Fig. 1A). As shown in Fig. 1B, the number of these cells was very limited, accounted for about 0.2%-0.5% of the total BM mononuclear cells. The number of living cell using the MACS method was slightly higher than the FACS method, suggesting that small nozzle diameters with high shear stresses have effect on cell viability. As a practical parameter from antibody-staining to the end of sorting, FACS procedures took 1.5-2 h, while manufacturer's protocol MACS procedures took approximately 25-30 min. Enriching CD133 + first by MACS before FACS could significantly shorten total isolation time from 4-6 h to 2-3 h.

### Morphological Structure of CD133 + /Lin-/CD45- Cells

**Fig. 2:**
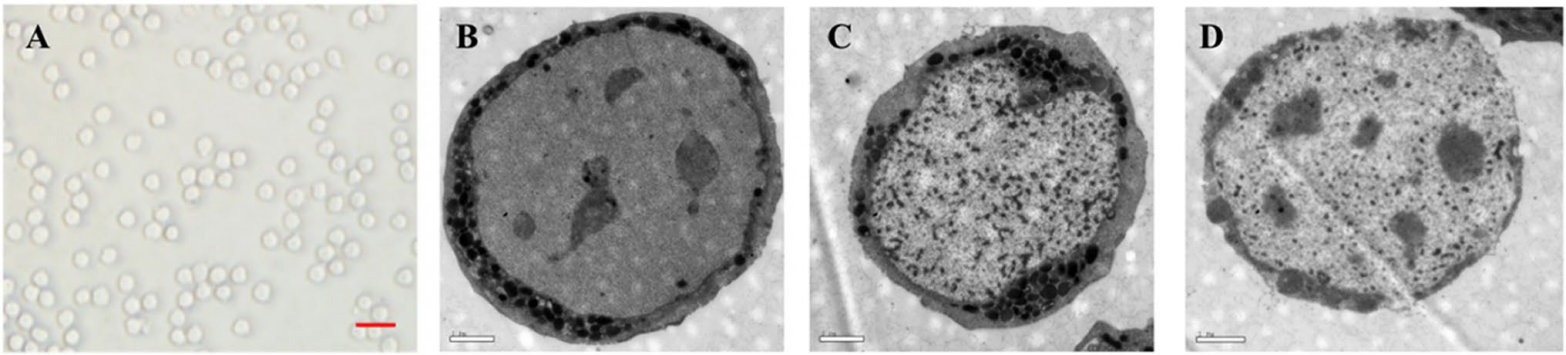
Morphological structure of CD133 + /Lin-/CD45- cells under inverted fluorescence microscope (**A**, 200x, Scale bars = 10um) and electron microscope (**B**-**D**, Scale bars = 1um). VSELs are small and measure 4-8 µm in diameter. They possess a relatively large nucleus surrounded by a narrow rim of cytoplasm. At the ultrastructural level the narrow rim of cytoplasm possesses a few mitochondria, scattered ribosomes, small profiles of endoplasmatic reticulum and a few vesicles. The nucleus is contained within a nuclear envelope with nuclear pores. Chromatin is loosely packed and consists of euchromatin

In the current study, we evaluated the morphology of these rare cells by employing transmission electron microscopy. We noticed that CD133 + /Lin-/CD45- cells were relatively small size, around 4-8 µm in diameter, contain relatively large nuclei and a narrow rim of cytoplasm. Most importantly DNA in the nuclei of these small cells contain open-type euchromatin that is characteristic for pluripotent stem cells (Fig. 2). Thus, we provide morphological evidence for the presence of CD133 + /Lin-/CD45- cells in swine BM for the first time.

### Culture and Amplification of CD133 + /Lin-/CD45- Cells

**Fig. 3:**
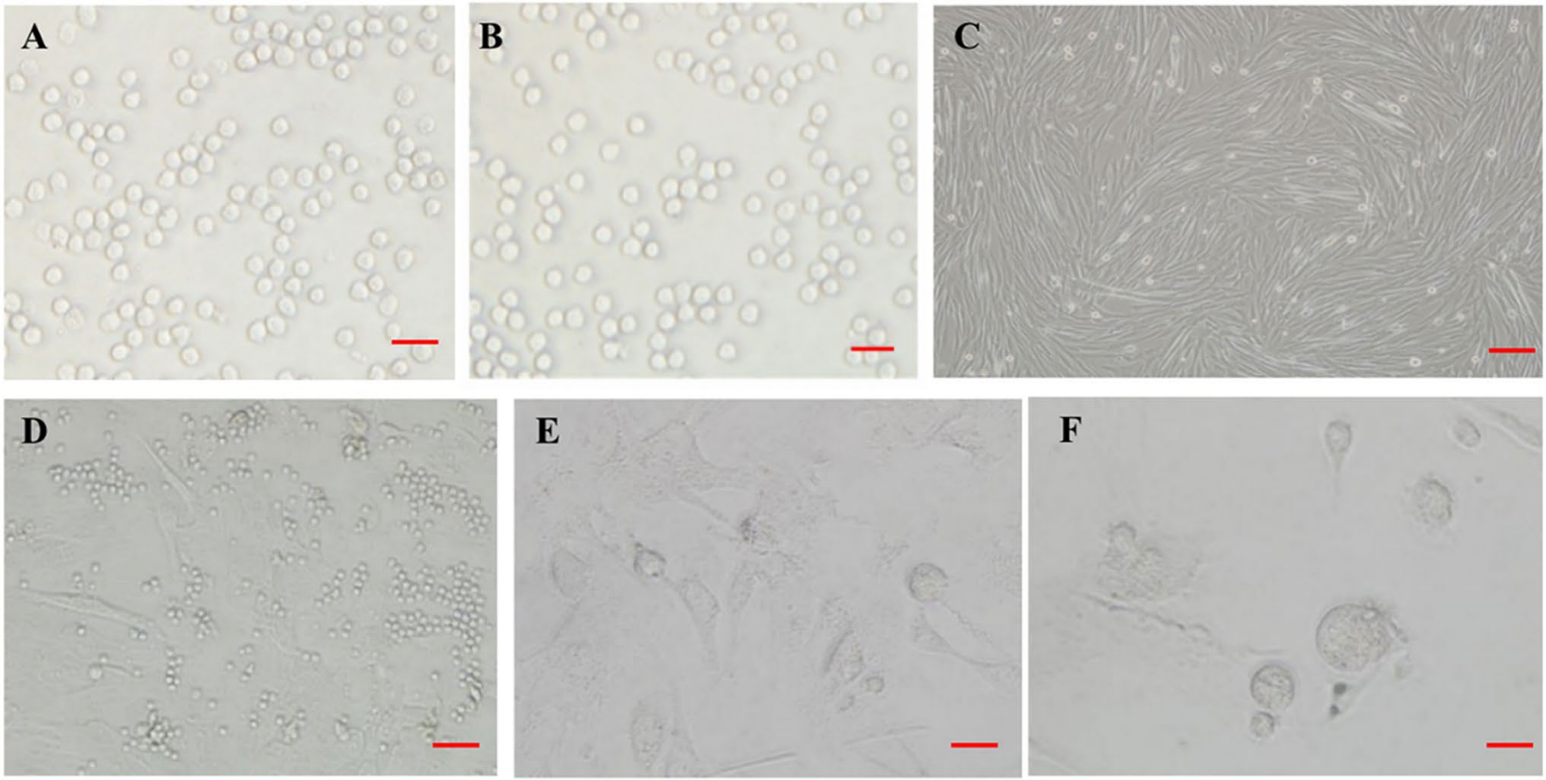
Culture and amplification of CD133 + /Lin-/CD45- cells. **A**-**B**: CD133 + /Lin-/CD45- cells could not proliferate in MSC and ES cell culture media, and they were floating as single cells. (100 × , Scale bars = 10um). **C**: Swine myoblasts feeding layer. (100 × , Scale bars = 10um). **D**-**F**: CD133 + /Lin-/CD45- cells were co-cultured with the feeding layer of myoblasts for 1d (D,100 × , Scale bars = 10um), 3d(E, 400 × , Scale bars = 1um), and 5d(F, 400 × , Scale bars = 1um), respectively. At 3d, the aggregation trend of cells could be observed under microscope. At 5d, small cell colonies similar to cell clones were formed

We simultaneously cultured BM CD133 + /Lin-/CD45- cells in both MSC and ES cell culture media. However, the BM CD133 + /Lin-/CD45- cells could not proliferate in these culture media, and they were floating as single cells. In order to promote the division and proliferation of CD133 + /Lin-/CD45- cells, the cells were cultured in culture plates with myoblasts cell feeder layer, together with 10% FBS and RPMI 1640. As shown in Fig. 3, a portion of the CD133 + /Lin-/CD45- cells proliferated and formed spheres that resembled ES cell-derived embryonic bodies on day 5 of culture.

### Identification of Stem Cell Marker

**Fig. 4:**
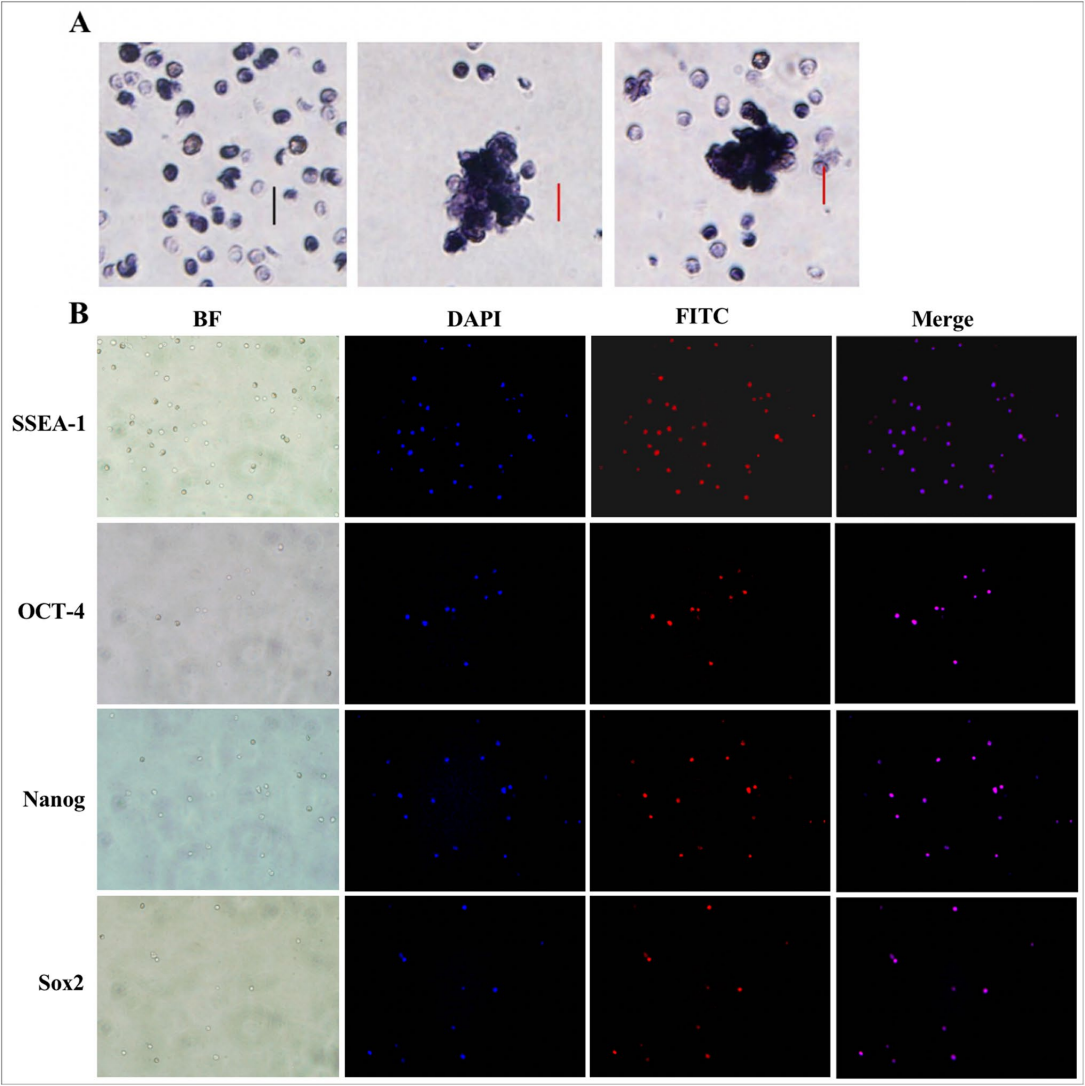
Identification of stem cell marker. **A**. AKP staining of CD133 + /Lin-/CD45- cells (left: single positive staining after isolation and purification; middle and Right: positive for cell clone staining in amplification culture; 100x, Scale bars = 10um); **B**. Confocal microscopic images of CD133 + /Lin-/CD45- cells. Isolated CD133 + /Lin-/CD45- cells were stained for SSEA-1, OCT-4 and SOX-2 (FITC, green fluorescence) and NANOG (PE-Cy3, red fluorescence). Nuclei were stained with DAPI (blue fluorescence)

The CD133 + /Lin-/CD45- cells expressed various cell surface markers which were specific for stem cells. Alkaline phosphatase (AKP) staining showed that both single cells and clones were positive (Fig. 4A). Immunofluorescence staining of stem cell antigens SSEA-1, SOX-2, OCT-4 and NANOG were positive (Fig. 4B). These results were consistent with the recovery of CD133 + /Lin-/CD45- cells in FACS analysis.

### Gene Expression Profiles of CD133 + /Lin-/CD45- Cells: Comparison with those of Myoblasts

**Fig. 5:**
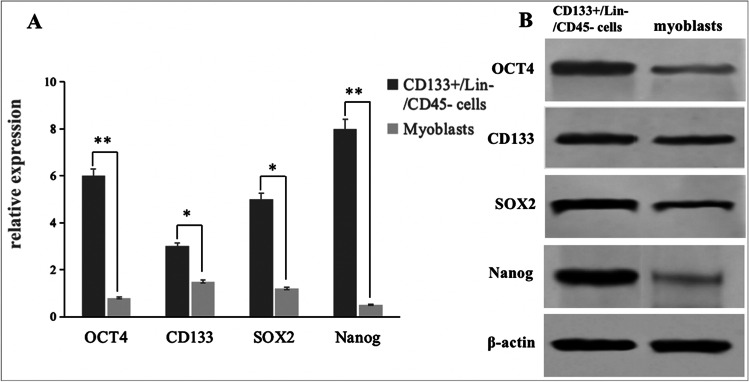
Expression of stem cell specific genes. **A**: Expression of mRNA for CD133、SOX2、OCT-4、CD45 and Nanog by RT-PCR in myoblasts and CD133 + /Lin-/CD45- cells. The graphs show the fold difference in concentration of mRNA for CD133、SOX2、OCT-4 and Nanog in sorted fractions when compared to myoblast. Results are presented as mean ± SD. Statistically significant differences (*P < 0.01, **P < 0.05) are shown when compared with myoblasts. Analysis was performed three times with samples prepared from two independent sorts. **B**: Gel electrophoresis results showed that CD133 + /Lin-/CD45- cells expressed CD133, Nanog, Oct4, Sox2

The gene expression profiles were analyzed using qRT-PCR to evaluate the expression of stem cell markers. As shown in Fig. 5, the gene expression profile of VSELs was unique, and was different from those of myoblast. Quantitative PCR and gel electrophoresis results showed that VSELs cells highly expressed stem cell specific genes CD133, SOX2, OCT4 and NANOG, while myoblasts only expressed β-actin (Fig. 5).

### Cardiac Fibrosis Rat Model Establishment

**Fig. 6:**
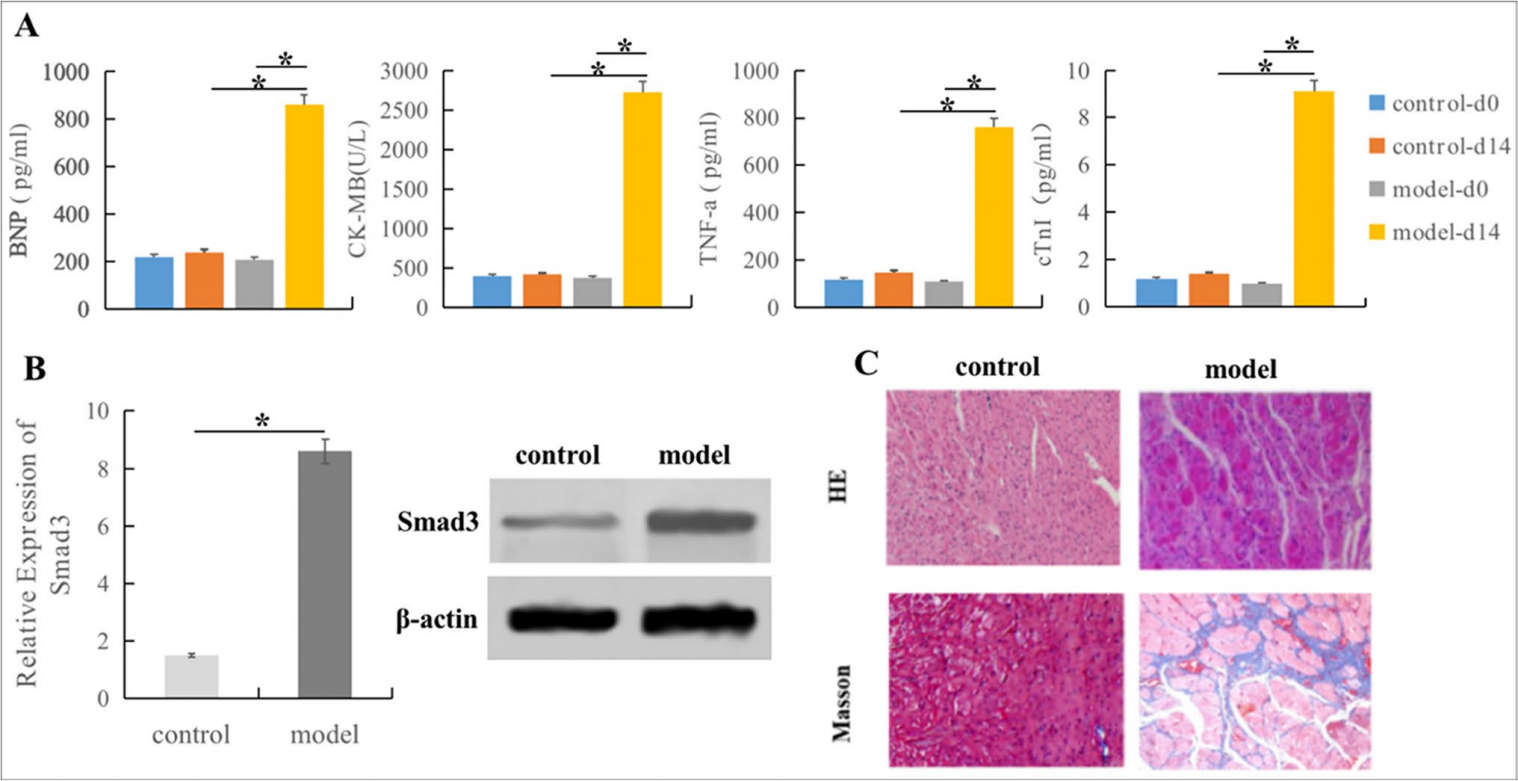
Establish of rat cardiac fibrosis models. **A**. The expression levels of myocardial injury indicators BNP, CK-MB, cTnI and inflammatory factor TNF-a were significantly increased in model group; *P < 0.05 vs. respective controls. **B**. qRT-PCR and WB showed that Smad3 expression increased in model group; *P < 0.05 vs. respective controls. **C**. HE staining and Masson staining indicated inflammatory cell infiltration, collagen fiber hyperplasia and focal myocardial necrosis in the model group

SD rats aged 4–6 weeks were fasted and anesthetized, and ALzet2002 200ul osmotic pump was inserted subcutaneously. Angiotensin II (Ang-II) diluted with acetic acid (1.44ug/g/d) was pumped into model group, and diluted acetic acid was pumped into control group. ELISA test of venous blood showed the levels of BNP, CK-MB, cTnI and TNF-a were significantly increased in the model group after 14 days. qRT-PCR and Western Blot showed that the expression of key gene Smad3 was significantly increased. HE staining and Masson staining showed inflammatory cell infiltration and increased collagen fiber, and a small number of large and deeply stained cells appeared. The abnormality of myocardium showed hyaline change, fissure-like fibrosis and collagen fiber hyperplasia. Some of them showed focal necrosis of myocardium, with abnormal nuclei and multinucleated cells, suggesting that CF model was successfully constructed in rats (Fig. 6).

### Isolation and Identification of Exo

**Fig. 7:**
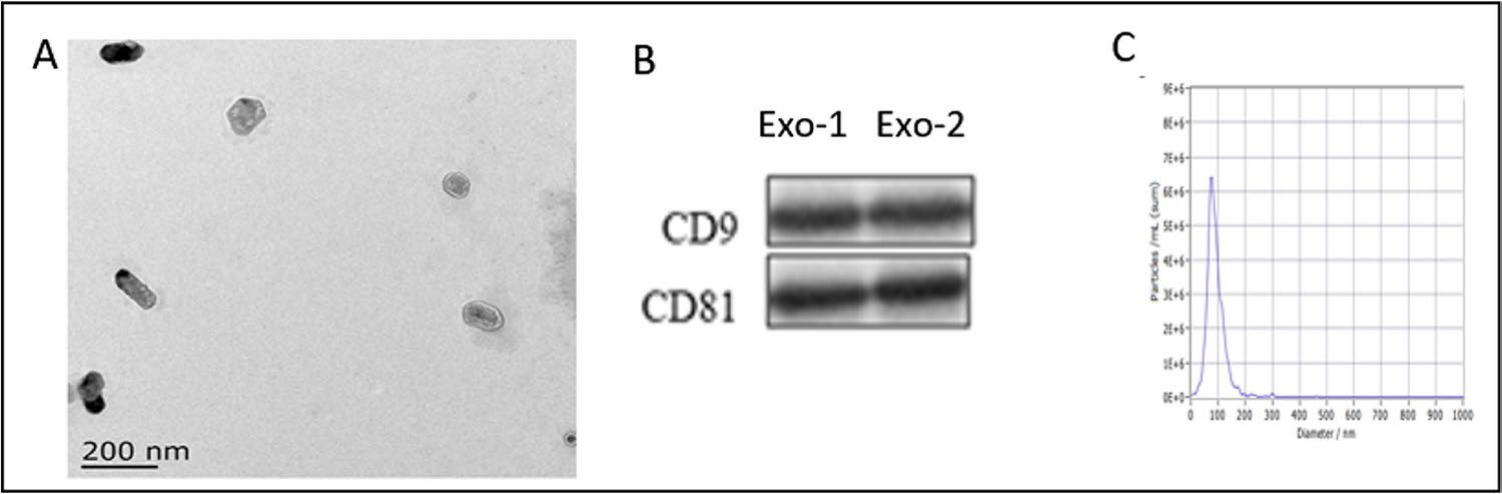
Isolation and identification of Exo. **A**. The morphology of Exos under electron microscopy. The diameter of an exosome is about 100 nm (Scale bar = 200 nm). **B**. Traditional exosomal markers, CD9 and CD81 were tested by Western blot in the Exos. **C**. The particle size distribution of the Exos by nanoparticle tracking analysis

We isolated exosomes from CD133 + /Lin-/CD45- cells and performed electron microscopy, western blotting, and nanoparticle tracking to identify the function of the exosomes. Electron microscopy showed isolated exosomes as short rod-like or round-shaped vesicles ranging from approximately 30 to 200 nm in diameter. Western blotting revealed that the exosomal preparations were enriched with the exosomal markers CD9 and CD81. Nanoparticle tracking analysis confirmed that the particle size of the Exos mainly ranged from 30 to 100 nm (Fig. 7).

### Reversible Effect of Exo on Cardiac Fibrosis

**Fig. 8:**
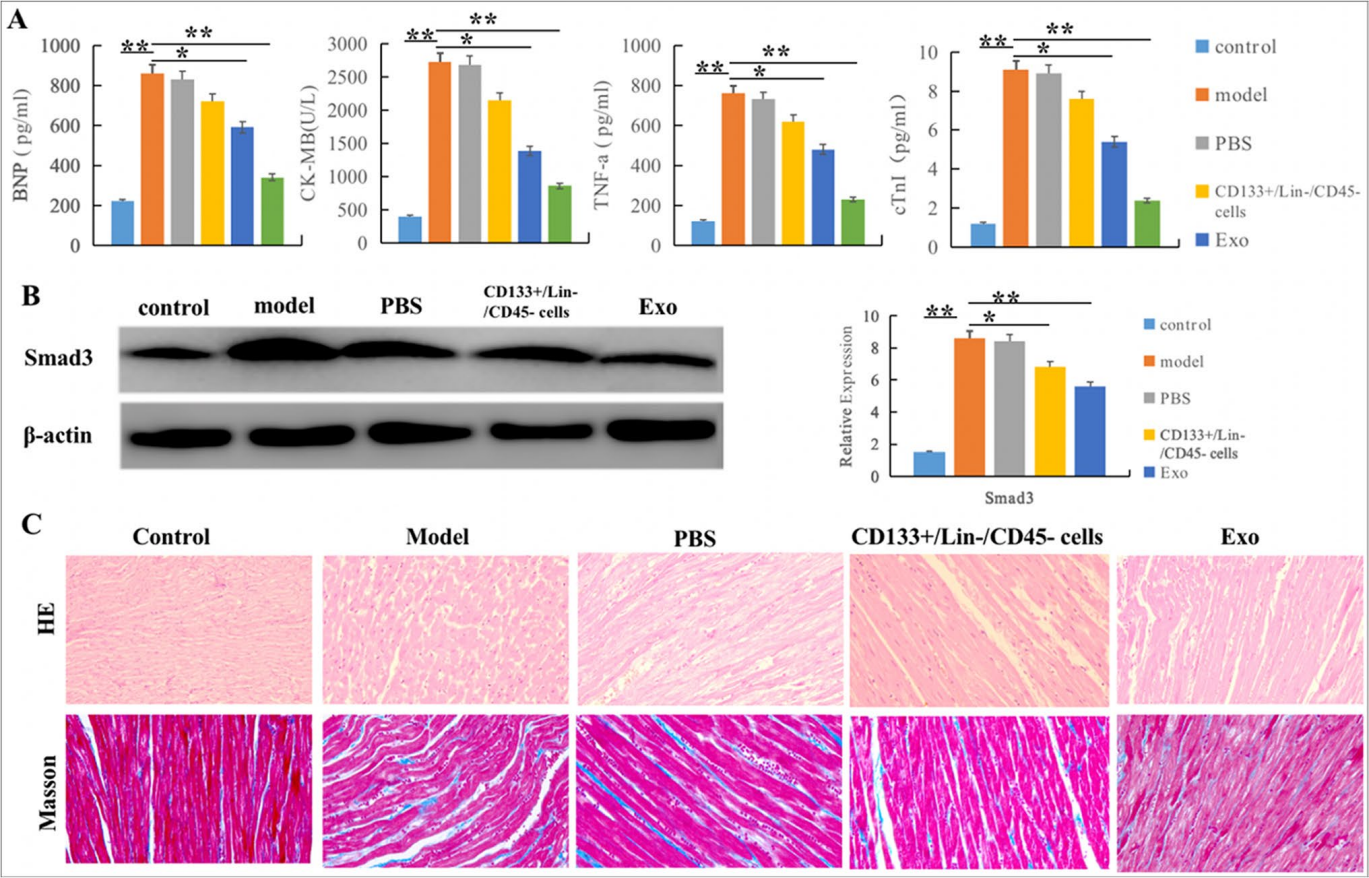
Reversible effect of Exo on cardiac fibrosis. **A**. ELISA showed that compared with model group, the levels of cardiac injury related indexes (BNP, CK-MB, cTnI and TNF-a) in Exo group were significantly decreased; **B**. RT-qPCR and Western Blot showed that compared with model group, mRNA and protein expression levels of Smad3 in Exo group were decreased; *P < 0.05, **P < 0.01 vs. control group. **C**. HE staining and Masson staining of heart tissue indicated that compared with the model group, the degree of myocardial cell necrosis and fibrous tissue proliferation were reduced after Exo treatment

Four weeks after Exo treatment, venous blood samples were collected for ELISA to detect myocardial injury related indicators (BNP, CK-MB, cTnI and TNF-a), qRT-PCR and Western Blot to detect the expression level of key gene Smad3, HE staining and Masson staining to evaluate the degree of myocardial fibrosis. The results indicated that the cardiac injury related indexes, the expression of key gene Smad3 and the degree of cardiac fibrosis in Exo treatment group were significantly reduced compared with the model group (Fig. 8).

**Fig. 9:**
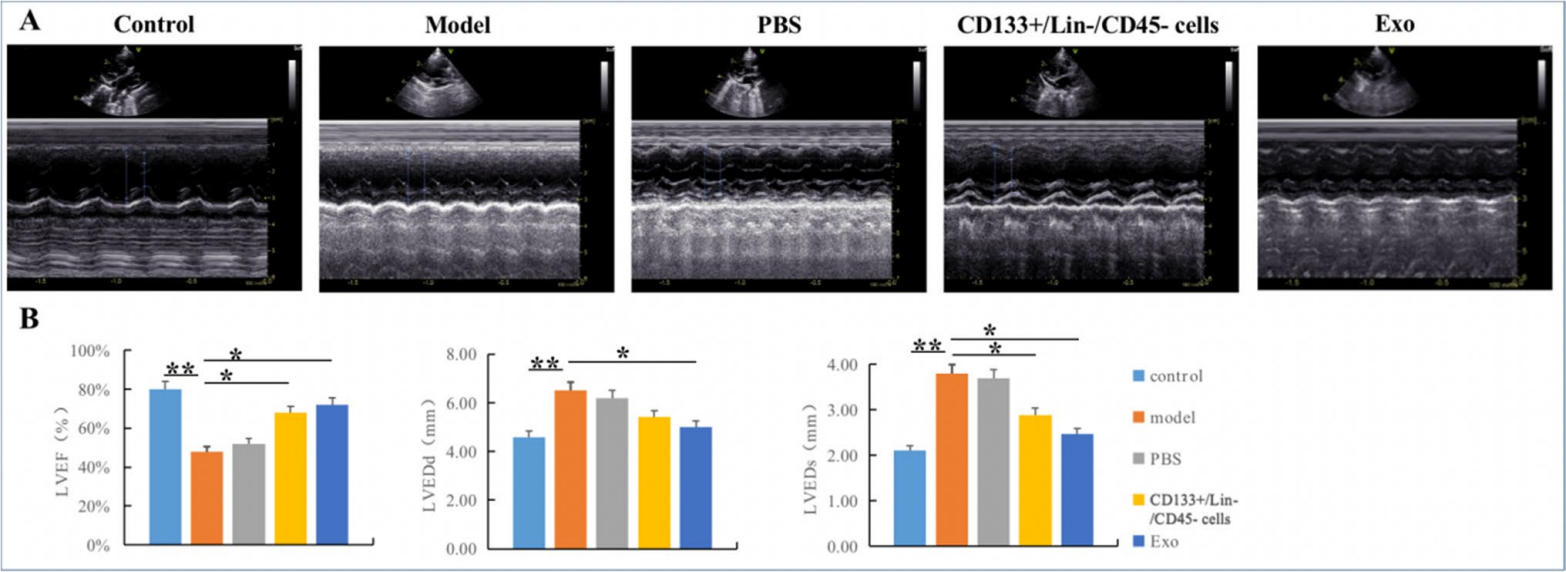
The cardiac function of rats measured by echocardiography. **A**. The typical image of 2D 2D echocardiography in each group. **B**. The value of LVEF, LVEDd and LVEDs in each group

The cardiac function of rats was measured by echocardiography (Fig. 9, Table 1). LVEDd and LVEDs of the rats in the model group were significantly increased, as compared with the control group (*p* < 0.01). EF were dramatically decreased (*p* < 0.01). Compared with the model group, LVEDd of the rats in the cells group were mildly decreased (*p* > 0.05), whereas the LVEDs were decreased and EF were increased significantly (*p* < 0.05). However, those value in the Exo group were significantly changed compared with model group (*p* < 0.05). These results demonstrated that Exo could improve heart functions which were compromised by Ang-II.

**Table 1:** Echocardiography assessment of the cardiac function in rats

Group	LVEF (%)	LVEDd (mm)	LVEDs (mm)
Control	82.31 ± 2.83**	4.67 ± 0.68**	2.18 ± 0.59**
Model	47.93 ± 9.94	6.52 ± 0.99	3.96 ± 1.21
PBS	51.04 ± 10.21	6.01 ± 1.41	3.77 ± 1.14
CD133 + /Lin-/CD45- cells	65.04 ± 12.69*	5.61 ± 0.81	2.89 ± 0.92*
Exo	74.89 ± 12.77*	5.02 ± 0.63*	2.47 ± 0.78*

Data represented the mean ± SD

^*^p < 0.05, ** *p* < 0.01 vs. corresponding model group

## Discussion

Evidence has accumulated that both murine and human adult tissues contain early-development stem cells with a broader differentiation potential than other adult monopotent stem cells [28, 29]. The nonhematopoietic stem cells appear to be heterogeneous and contain cells committed to mesenchymal and endothelial lineages, as well as more primitive multipotential cells resembling progenitors of germ cells and very small embryonic-like stem cells (VSELs) [30]. Although tremendous progress has been reached in mouse, the physiological traits of rodents are far apart from human. Swine and human are highly homologous, which are treated as the optimal organ donor. The identification of VSELs residing in swine bone marrow may expedite possible applications of this intriguing cell in regenerative and precision medicine.

In order to develop safe and efficient regenerative therapies, appropriate amount of pluripotent autologous cells is necessary. So far, only embryonic stem cells (ESCs) and induced pluripotent stem cells (iPSCs) are proven to be pluripotent, giving rise to all the cells from the three embryonic germ layers. However, the application of ESCs in clinic therapy is subject to ethic issue and their inherent tumorigenesis[31]. The creation of iPSCs can overcome these drawbacks, since they are proposed to be identical to ESCs–regarding to the functionality. Meanwhile, the application of iPSCs is limited by the low reprogramming efficiency to produce these cells. Further more, the use of iPSCs in therapies is restricted due to their methylation profile, the applied reprogramming methods, and genetic modifications are biological hazard [32, 33].

A rare Sca1 + Lin − CD45 − SCs population were initially identified, isolated from adult mice via fluorescence activated cell sorting (FACS) and named as VSELs [34]. VESLs were furtherly isolated from human cord blood [35], several adult murine tissues and organs [36]. Although VSELs are currently studied in a lot of laboratories worldwide, the series researches were mainly contributed by Kucia & Ratajczak and their colleagues [37]. Recent reports proposed that VSELs have similar characteristics as ESCs and could serve as basis for therapeutic applications. VSELs possess very primitive morphology and express PSCs markers [26](e.g. Oct4, Nanog and SSEA-4) as well as the surface phenotype Sca1 + /CD133 + Lin − CD45 − in mice/humans. As VSELs can be mobilized into peripheral blood following acute MI [38], improve heart function and alleviate cardiac remodeling [39], these cells seem to possibly become an optimal seed cells for cardiovascular regeneration medicine. Recently, employing anti-CD133-conjugated paramagnetic beads followed by staining with Aldefluor has also been proposed for a faster large-scale VSELs isolation [40]. More recent evidences demonstrate that VSELs deposited in adults tissue share several markers with epiblast/germ line cells and are responsible for tissue regeneration after organ injuries in rejuvenation of the TCSCs. Recent reports proposed that VSELs coming from primordial germ cells, give rise to hematopoietic stem cells (HSCs), and endothelial progenitor cells (EPCs) and are a source of mesenchymal stem cells (MSCs) and TCSCs [41]. As a promising candidate, their unique characteristics and potentiality may contribute to myocardial and endothelial regeneration.

This is the first study to establish the method of identification and isolation of the BM CD133 + /Lin-/CD45- VSEL cells, to explore its optimized sorting process and observe the micro-morphological structure of swine BM VSELs in vitro. For experimental animals, we conducted above procedures with adult (12-18 m), young (3-6 m) and newborn (< 3d) swines successively. Although a large amount of bone marrow (50-100 ml) could be extracted from adult and young swines in each experiment, the proportion of VSELs in bone marrow fluid was very low. The number of VSELs that were finally isolated and purified was very small (about 1–5 × 10^4^ cells). Meanwhile, from newborn swines, only about 10 ml bone marrow was isolated in each experiment, but the proportion of VSELs was very good, and the amount of VSELs finally got was up to 1–5 × 10^6^ cells. These results suggest that the proportion of VSELs in the bone marrow decreases rapidly with aging, which is consistent with other previous studies [42].

Recent evidence indicates that adult murine BM harbors a multitude of non-hematopoietic stem and progenitor cells in addition to well described HSCs [43]. Such SC populations belonging to non-hematopoietic compartment of BM tissue include MSC [44] and VSELs. BM-derived murine VSELs have been isolated and characterized based on surface antigens as well as gene expression as a population of Sca-1^+^/Lin^−^/CD45^−^/Oct-4^+^/Nanog^+^ cells. Importantly, several groups have reported the existence of such SCs in other murine and human tissues including ovaries and testes [45, 46].

In this study, we isolated and antigenically defined a population of stem cells with developmentally early characteristics in swine BM. The very small size of VSELs requires optimized strategy for sorting. It was reported that the proportion of BM VSELs in BM-derived nucleated cells (BMNCs) is very low (0.2%-0.6%) [47]. Therefore, it was difficult to efficiently isolate VSELs because of their rarity, which may be the major factors of the recent controversy surrounding the existence of VSELs. To isolate VSELs from the BM by FACS, we employed a novel size-based efficient approach controlled by size bead markers for isolating Lin^−^/CD45^−^/Sca-1^+^ VSEL phenotypic cells identified in swine BM. We tested and optimized protocol for sorting VSELs by flow cytometry. Erythrocyte lysis buffer is used instead of Ficoll centrifugation because the latter might deplete the population of very small cells [48]. Our results were consistent with a previous report from Wojakowski W [49].

VSELs morphology and ultrastructure have also been described indicating their very small size (smaller than erythrocytes) and higher N/C ratio which supports their primitive nature. Swine BM VSELs were characterized by small size, large nucleus, small amount of cytoplasm, expression of PSC transcription factors and ability of trans-reproductive differentiation. Human BM VSELs contains a population of CXCR4^+^/CD34^+^/CD133^+^/Lin^−^/CD45^−^. BM-derived murine VSELs have been isolated and characterized based on surface antigens as well as gene expression as a population of Sca-1^+^/Lin^−^/CD45^−^/Oct-4^+^/Nanog^+^ cells. Several groups have reported the presence of VSELs in other murine and human tissues including bone, ovaries and testes. Previous studies have reported presence of pluripotent cells expressing OCT-4, NANOG and regulate the proliferation and development of VSELs [50]. BM VSELs expressed CXCR4, suggesting that the SDF-1/CXCR4 axis plays a role in the homing of BM VSELs into the BM [51]. VSELs’ morphology expressed unique molecular characteristics of ESCs’ pluripotency, which can be induced into three germ layers.

Some reports indicated that there were VSELs in testis and ovary, which are featured with MSCs. Discovered in 1970 [52], MSCs possess several specific features that make them important candidates for future regenerative cardiac therapies. MSCs play an important role in bone defects, not only because of their multipotency but also as a source of cytokine supply. For adult SCs, mainly MSCs, translation from bench to clinical trials dates back to the late 1990s and early 2000s, when evidence for multipotentiality of MSCs was published by Osiris Therapeutics [53] and the first clinical trials were performed using MSCs [54]. MSCs have been shown to be safe and to promote heart function improvements [55]. However, the studies have been demonstrated that the differentiation ability of BM derived mesenchymal stem cells (BM-MSCs) decreased with increasing age.

It is likely that the interaction between these cells and the transplanted MSCs play a role in recovery of fertility. Moreover, these BM VSELs were able to differentiate into cardiomyocytes(mesoderm); neurons, astrocytes, and oligodendrocytes (ectoderm); and pancreas (endoderm) in co-culture systems. However, these pluripotent stem cells are autologous, embryo-free and potentially safe for regenerative medicine with no associated ethical issues as compared to ESCs [13]. VSELs could be mobilized into PB when organ and tissue injury and circulate there in an attempt to regenerate damaged organs [56]. In vivo studies have shown that cells differentiated from SCs can repair damaged organs, but repairs are a bit powerless for a large number of damaged tissues [57]. This physiological mechanism probably plays a more significant role in the regeneration of small tissue and organ injuries, for example, the regeneration for pacemaker cell.

Working on VSELs for almost 6 years, our team has found that the reasons for the differences observed in detection of VSELs and why they remain poorly studied until now. The most important thing is to overcome the technical hurdles to detect them. At first, a study showed that VSELs from human UCB lack SCs characteristics and fail to expand in vitro under a wide range of culture conditions [58]. Similar to the controversies surrounding protocols to detect VSELs, recently four independent groups completely negated the existence of ovarian stem cells (OSCs) on technical grounds [59]. But this does not imply that VSELs or OSCs do not exist. Rather, two distinct populations of stem cells exist in adult ovary surface epithelium (OSE) including relatively quiescent VSELs and active OSCs [60]. The presence of two stem cell populations (including active and dormant) in various adult organs has also been demonstrated by other teams [61].

Importantly, this study opens new perspectives for researching VSELs in swine tissues and organs in normal conditions as well as for examining their potential regenerative capacity in several unique swine tissue injury models. This would provide a new impact on current knowledge about VSELs including potential regenerative capacity of these unique SCs, which still need to be investigated in vitro as well as in vivo studies. Until now, the biological characteristics and role of VSELs were studied mostly in mice and human. No information of VSELs from large animal close to human has been reported yet. Future clinical studies using autologous VSELsneed those large animal experimental data. Swine and human are highly homologous, which are treated as the optimal organ donor. The identification of VSELs residing in swine bone marrow may expedite possible applications of this intriguing cell in regenerative and precision medicine. According to our primeval experience, the cell extraction efficiency from piglets is higher than adult swines. So piglet (< 3d after birth) may be an optimal model for the future research.

Earlier studies suggested stem cells could be used to treat tissue damage and fibrosis because they can migrate to the site of injury and differentiate into cells needed for tissue repair. Gao et al. injected MSCs intravenously into the rat model of radioactive myocardial injury and found that both CF level and cardiac function were improved [62]. Santoso et al. also found that MSCs transplantation can improve cardiac function after myocardial infarction by regulating autophagy of hypoxia-damaged myocardial cells [63]. However, at the same time, studies have shown that stem cells have a low survival rate in the inflammatory, ischemic and hypoxic environment, and only 1% of stem cells can survive more than 4 days after transplantation [64, 65]. The low number of stem cells may not be enough to play a therapeutic role. In recent years, studies have found that a variety of cells can secrete Exo, which contains a variety of active substances and can participate in the regulation of biological processes such as cell proliferation, differentiation and apoptosis. Repair of damaged tissues by Exo can avoid problems such as low survival rate, immune rejection and tumorigenesis in cell therapy [66]. SHAO et al. injected MSCs-Exo into the rat model of myocardial infarction, and found that the inflammatory response and fibrosis levels of rat myocardium were improved and the cardiac function was also enhanced [32]. It has also been found that Exo derived from macrophages can also reduce the fibrosis level after myocardial infarction by inhibiting the hyperactivation and proliferation of fibroblasts [67]. Exo derived from embryonic stem cells and adipose mesenchymal stem cells has also been confirmed to improve cardiac dysfunction caused by myocardial infarction by reducing inflammatory response, apoptosis and CF, respectively [68, 69]. Our study proved that VSELs-Exo could reduce inflammatory response, reduce collagen fiber deposition, reduce injury area and improve cardiac function. Therefore, we speculated that the inhibiting effect of VSELs on CF may mainly depend on Exo secreted, but what kind of small molecule substances in Exo are involved in CF repair and the specific mechanism need to be further explored.

Fang et al. found that Exo derived from umbilical cord mesenchymal stem cells carries a group of specific mirnas (Mir-21, Mir-23a, Mir-125b and Mir-145) that can directly regulate the expression of TGF-β2, TGF-βR2 and Smad2 in the TGF-β/Smad2 signaling pathway. They can inhibit fibroblast activation, reduces α-SMA expression and type I collagen deposition, and alleviates tissue fibrosis [70]. Lima Correa B et al. found that EV-CPC do not trigger cardiomyocyte proliferation but still improve cardiac function by other mechanisms which may include the regulation of fibrosis [71]. Kervadec A et al. found that either hESC-Pg or their secreted EV enhance recovery of cardiac function and similarly affect cardiac gene expression patterns that could be related to this recovery [72]. Khan et al. found that Exo derived from embryonic stem cells are rich in mirNA-290–295 gene clusters, which can enhance the survival and proliferation of cardiac progenitor cells and reduce CF [73]. Gray et al. found that hypoxia preconditioning up-regulated the expression of 11 mirnas in cardiac progenitor cell derived Exo, and treatment of mouse fibroblasts with this Exo significantly inhibited the expression of TGF-β and type I /III collagen and reduced the degree of fibrosis [74]. These studies suggest that stem cell-derived Exo can deliver miRNA into target cells to regulate gene expression and participate in the CF repair process.

In addition, pharmacological intervention can alter the content composition of stem cell Exo and influence its biological effects [35, 36]. Ruan et al. found that The MSCs pretreated with Suxiao Jiuxin Pill could promote the proliferation of myocardial cells by increasing the level of histone 3 lysine 27 trimethylation, and could also mediate the gTPase-dependent pathway to increase the synthesis and secretion of Exo in MSC by up-regulating the expressions of Rab27a, SYTL4 and Rab27b. Thus enhancing the therapeutic efficacy of Exo [75, 76]. Our study found that treating VSELs with 5-AZA could enhance the therapeutic effect of Exo on CF. In this sense, the findings of our study are expected to reveal the molecular mechanism of CF repair from the perspective of drug combination with VSELs-Exo, and provide a target for the development of new therapies for heart failure.

A limitation of our research is the small number of swine studied. We found that it is very difficult for VSELs to be cultured or expanded in vitro using general culture conditions. Thus, further investigation is required to determine whether these cells are merely developmental remnants found in the adult tissue that cannot be harnessed effectively for regeneration medicine. Furthermore, it has seldom obtained for parallel experiments to compare several populations of putative SCs to assess the similarities and differences between these cell populations.

## Conclusions

In conclusion, the importance of SSEA-1^+^Oct-4^+^Sca-1^+^/CD133^+^CXCR4^+^Lin^−^CD45^−^ pluripotent VSELs in adult tissue or UCB is now being stressed. New data has provided mounting evidence on the existence and potential biological role of VSELs. VSELs may be a promising PSCs population for future clinical application. Their regenerative potential should be confirmed in large animal model similar to humans and technical issues regarding their isolation, expansion and differentiation need to be furtherly addressed. We also look forward to sharing the procedure of isolation and expansion of these rare cells, as well as to learning their differentiation potential in vitro and in vivo. More in-depth researches may shed a light on the clinical application for regenerative medicine and precision medicine.

## Data Availability

All data generated or analyzed during this study are included in this published article.

## Abbreviations

VSELs: Very small embryonic-like stem cells
Exos: Exosomes
BM: Bone marrow
CF: Cardiac fibrosis
MACS: Magnetic Activated Cell Sorting
FACS: Fluorescence-activated cell sorting
ESCs: Embryonic stem cells
iPSCs: Induced pluripotent stem cells
BM-MNCs: Bone marrow-derived mononuclear cell
UCB-SCs: Umbilical cord blood-derived stem cells
TCSCs: Tissue committed monopotent stem cells
